# Identification and Characterization of a Novel Microvitellogenin from the Chinese Oak Silkworm *Antheraea pernyi*

**DOI:** 10.1371/journal.pone.0131751

**Published:** 2015-06-30

**Authors:** Yanqun Liu, Miaomiao Chen, Junfang Su, Hongfang Ma, Xixi Zheng, Qun Li, Shenglin Shi, Li Qin

**Affiliations:** 1 Department of Sericulture, Shenyang Agricultural University, Shenyang, Liaoning, China; 2 Sericultural Institute of Liaoning Province, Fengcheng, Liaoning, China; 3 School of Basic Medicine, Guangzhou University of Chinese Medicine, Guangzhou, Guangdong, China; Uppsala University, SWEDEN

## Abstract

Microvitellogenin (mVg) is a relatively small vitellogenic protein only characterized in the eggs of the lepidopteran insects *Manduca sexta* and *Bombyx mori*. In the present study, we report a novel mVg (ApmVg) isolated from the Chinese oak silkworm *Antheraea pernyi*. The obtained *ApmVg* cDNA sequence contains an open reading frame of 783 bp encoding a protein of 260 amino acids with a predicted molecular weight of 29.96 kDa. This gene does not contain introns. Structural analysis revealed that this protein shares putative conserved domains with the lepidopteran low-molecular weight lipoprotein, which belongs to the lipoprotein_11 superfamily. The protein sequence of ApmVg exhibits 48% sequence identity with mVg from *M*. *sexta* and 40–47% sequence identity with the 30K lipoproteins from *B*. *mori*. Phylogenetic analysis suggests that ApmVg is a novel member of the lepidopteran low-molecular weight lipoproteins. Transcriptional analysis indicated that *ApmVg* mRNA is mainly expressed in the fat body (both female and male) during post-diapause development of the pupal stage, and it was also detected in ovaries and spermaries in smaller amounts. RT-PCR and Western blot analyses revealed that ApmVg is synthesized by the fat body and secreted into hemolymph and ultimately accumulates in eggs. The *ApmVg* transcript can be detected in the fat bodies of female pupae four days after treatment with 20-hydroxyecdysone and shows an expression pattern distinct from that of *vitellogenin *(*Vg*), which is detectable throughout diapausing and in post-diapause development. ApmVg decreased dramatically during embryonic development. These results represent the first study of mVg outside *M*. *sexta* and *B*. *mori* and provide insight into the physiological role and evolution of mVgs.

## Introduction

In animals, yolk proteins are essential to ensure a sufficient supply of nutrients for the developing embryo [1]. The major yolk protein, vitellin, is derived from the precursor vitellogenin (Vg). Vgs are large molecules (approximately 200 kDa) synthesized by the fat body, secreted into the hemolymph, and transported to the developing oocytes. The Vgs have been extensively studied in animals ranging from vertebrates to invertebrates [2,3].

Microvitellogenin (mVg) is a relatively small vitellogenic protein with a molecular weight of approximately 30 kDa. mVg was first identified in *Hyalophora cecropia* [4] and was initially known as “microvitellin”. Later, a female-specific hemolymph protein with properties similar to those of the *H*. *cecropia* protein was isolated from *Manduca sexta* and named “microvitellogenin” [5,6]. In *M*. *sexta*, mVg comprises approximately 6% of the soluble egg proteins [6]. mVg is a female-specific yolk protein in moths, and *mVg* mRNA is only present in the adult female fat body [7]. This protein accumulates in the eggs, presumably via an endocytic process, but mVg does not use the same endocytic receptor as that of Vg [8].

A close evolutionary relationship between mVg from *M*. *sexta* and typical 30K proteins from several lepidopteran insects, including *Bombyx mori*, has been demonstrated [9–12]. 30K proteins are classified into the lepidopteran-specific lipoprotein_11 family. In *B*. *mori*, 30K proteins comprise approximately one-third of the total embryo yolk proteins [13] and progressively accumulate in the hemolymph from the fifth instar larvae to moth stages [12,14,15]. The 30K proteins are synthesized by the fat body, secreted into the hemolymph, transported into eggs, and degraded in the embryonic gut lumen [12].

In the present study, we report a novel mVg from the Chinese oak silkworm *Antheraea pernyi* (Lepidoptera: Saturniidae). This species is an economically important insect used for silk production and as a source of insect food for human consumption [16,17]. We examine the developmental expression profile of ApmVg and show that it is synthesized by the fat body and ultimately accumulates in the eggs. We also show a distinct expression pattern between the *mVg* gene and the *Vg* gene. Our data could help delineate the physiological functions of these related proteins.

## Materials and Methods

### Insect

The *A*. *pernyi* strain *Shenhuang No*. *2* was maintained in our laboratory. The pupae were kept at room temperature until entering winter diapause. The diapausing pupae were kept at 5°C for 50 days to terminate diapause, and the diapause-activated pupae were then transferred to 25°C until moth emergence. The whole female and male pupae were collected when the developing ovarian follicles undergoing vitellogenesis had formed in the female pupae. During the pupal developmental stage, the fat body of female pupae was collected at various periods, including pre-diapause, diapause, post-diapause 1 (without developing ovarian follicles), and post-diapause 2 (with developing ovarian follicles). The female pupae in post-diapause 1 were used to obtain samples of the hemolymph, fat body, ovary, spermary, and brain. Eggs at day 5, whole larvae at day 10 of the fifth instar, and adults were also sampled. 20-Hydroxyecdysone (20-E) was used to initiate the development of diapause pupae, and 100% of the treated pupae emerged as moths within 14–20 days at 25°C. Then, the fat bodies and ovaries of 20-E-treated pupae were dissected at various time points to compare the temporal expression profiles of the related genes.

### Genomic DNA extraction, total RNA extraction, and first-strand cDNA synthesis

Genomic DNA was extracted from a single egg at day 5 using the TIANamp Genomic DNA Kit (TIANGEN Biotech Co., Ltd., Beijing, China). Total RNA was extracted using the RNAprep pure Tissue Kit (TIANGEN Biotech Co., Ltd.). Using 2 µg of total RNA per sample, the first-strand cDNA was generated using the oligo(dT)_15_ primer with the TIANScript RT Kit (TIANGEN Biotech Co., Ltd.).

### Gene isolation and sequence analysis

A pupal cDNA library of *A*. *pernyi* has been constructed in our laboratory [18], and randomly selected positive clones were sequenced by the expressed sequence tag (EST) method [19]. An EST encoding the homologue of mVg from *M*. *sexta* was identified. Thus, the cDNA clone was used to complete the full-length cDNA sequence. To identify the open reading frame (ORF), deduce the amino acid sequence, and predict the isoelectric point and molecular weight of the deduced amino acid sequence, DNASTAR software (DNASTAR Inc., Madison, Wisconsin, USA) was used. The entire ORF of this gene was successfully amplified with the primer pair ORF-F and ORF-R (Table 1) and confirmed by sequencing. The cDNA sequence was compared with other homologue sequences deposited in GenBank using the “BLAST-X” tools at the National Center for Biotechnology Information (NCBI) web site. Conserved domains of the amino acid sequence were predicted at http://www.ncbi.nlm.nih.gov/Structure/cdd/wrpsb.cgi/. The deduced amino acid sequence was used to predict the protein signal peptide with the SignalP server on-line tool (http://www.cbs.dtu.dk/services/SignalP/). Subcellular localization predictions were performed at http://www.bioinfo.tsinghua.edu.cn/SubLoc/. The transmembrane protein topological structure was analyzed with the TMHMM server on-line tool (http://www.cbs.dtu.dk/services/TMHMM/). A motif scan was performed at http://hits.isb-sib.ch/cgi-bin/motif_scan.

**Table 1:** Primers for RT-PCR used in this study.

Gene	Forward primer	Reverse primer	PCR product (bp)	References
**Full ORF**				
*mVg*	ORF-F: ATGGG CCTGT CGCCT TTCGT	ORF-R: TTAGA AAGGT GCGAT AAACC ATC	783	This study
**RT-PCR**				
*mVg*	LYQ124: GTGGC TCGGA CACGC TATTT	LYQ125: ACGCC AACCT ATCTC CCACA	268	This study
*Vg*	LYQ156: CGAGA ACAGC GACCC GAAGA	LYQ157: GGCAA TGTGA CTGGC AACCT	290	[19]
*KK-42BP*	LYQ83: CCGCT CTCAT AGTAA AAACA A	LYQ84: AGGTC CTAAC AGTAG CCAGT C	448	[20]
*eIF-4A*	LYQ205: TCCAT CGCTC AGGCT GTTAT	LYQ206: GTGCT CGTCT GTCAC TTTCA	340	[21]
**qRT-PCR**				
*mVg*	F: GCCAATGAAGATGATACT	R: CACGGTTGAAGATATAGAA	88	This study
*eIF-4A*	F: TCCTC TCGTG TGCTT ATC	R: CCACC TCTTC CGATT CTAT	128	[21]

### RT-PCR analysis

The gene-specific primer pairs for *mVg*, *Vg* [20], and *KK-42BP* (*KK-42-binding protein*; [21]) are listed in Table 1. The *eIF-4A* gene was used as an internal control [22]. We used the reverse transcription-polymerase chain reaction (RT-PCR) method to detect the expression pattern. RT-PCR amplification was performed in a total reaction volume of 25 µl containing cDNA template, 1× PCR buffer, 10 pmol of each primer, 0.25 mM dNTP, and 2.5 units of Taq DNA polymerase (TIANGEN Biotech Co. Ltd.). PCR was performed with the following protocol: initial denaturation at 95°C for 3 min; 30 cycles of 45 s at 95°C, 30 s annealing at 55°C, and 30 s extension at 72°C; and a final extension at 72°C for 7 min. The amplification products were analyzed on 1.0% agarose gels stained with ethidium bromide. The RT-PCR experiments were performed three times. The RT-PCR products were purified from the gel and sequenced to confirm their specificity.

For quantitative RT-PCR analysis, the specific primer pairs for *mVg* and *eIF-4A* were designed using Beacon Designer 7.0 software (Premier Biosoft International). The qRT-PCR was performed using a Roche Light Cycler 480 (Hoffmann-La Roche Ltd.) with the following protocol: initial denaturation at 95°C for 2 min; 40 cycles of 15 s at 95°C, 30 s annealing at 60°C, and 30 s extension at 68°C; and a 60–95°C melting curve to analyze the amplified products. The relative changes for gene expression were calculated using the 2^-ΔΔCt^ method [23]. The qRT-PCRs were performed three times in parallel for each cDNA sample from independent RNA extractions. Statistical analysis was performed with SPSS 16.0. Two-tailed Student’s test was used to determine the difference between the groups, and P<0.01 was considered significant.

### Homologous comparison and phylogenetic analysis

Using the deduced protein sequence of *A*. *pernyi* mVg as the query, a BLAST search was performed at http://www.ncbi.nlm.nih.gov/blast.cgi. The protein sequences of mVg homologues from other organisms were retrieved from the available databases, including GenBank, EMBL, and other databases. Clustal X software was used to generate amino acid sequence alignment [24]. The phylogenetic tree was constructed by MEGA version 5.0 [25] using the Maximum Likelihood (ML) method. The WAG+G model was selected as the best model. The statistical significance of a group in the tree was assessed by the bootstrap probability with 1,000 replications.

### Antibody preparation, SDS-PAGE, and Western blotting

A 15-mer peptide (TRYFENENKGEIVEE) from *A*. *pernyi* mVg was designed and synthesized (CoWin Bioscience Co., Ltd, Beijing, China). This synthetic peptide was conjugated to bovine serum albumin (BSA) as a carrier protein. This conjugate in phosphate buffered saline (PBS) emulsified with Freund’s complete adjuvant was injected into a New Zealand White rabbit. Polyclonal antiserum was collected after the last immunization and stored at –80°C. Protein samples were mixed with 4% SDS and 2% β-mercaptoethanol, denatured at 100°C for 5 min, and separated on tricine sodium dodecyl sulfate-polyacrylamide gel electrophoresis (SDS-PAGE) using a 10% acrylamide gel. The gel was stained with Coomassie brilliant blue R-250. By an electrophoretic transfer system, protein samples were then transferred onto a polyvinylidene difluoride (PVDF) membrane. The membranes were blocked with PBS containing 0.1% Tween-20, incubated with anti-mVg antibodies, washed with PBS containing 0.1% Tween-20, and incubated with horseradish peroxidase (HRP)-conjugated sheep anti-rabbit IgG antibody. The final detection was performed with an HRP-DAB detection kit (CoWin Bioscience Co., Ltd, Beijing, China).

## Results

### Sequence characteristics of *A*. *pernyi* mVg

By EST analysis, a gene encoding mVg was identified from *A*. *pernyi*. The obtained 928 bp cDNA sequence contains a 5’-untranslated region (UTR) of 40 bp, a 3’ UTR of 105 bp with a polyadenylation signal sequence AATAAA at position 824 and a poly (A) tail, and an ORF of 783 bp encoding a polypeptide of 260 amino acids (S1 Fig). The deduced protein has a predicted molecular weight of 29.96 kDa and an isoelectric point of 4.99. Blastp search revealed that the protein sequence shared 48% (127/158) identity and 69% (180/258) positive residues with mVg from *M*. *sexta* (AAA29342; [9]). Therefore, we refer to this protein as *A*. *pernyi* mVg. This cDNA sequence has been deposited in GenBank under accession no. KM926620.

Conserved domain prediction showed that this protein shared putative conserved domains with the lepidopteran low-molecular weight lipoprotein also known as 30K lipoprotein and belonged to the lipoprotein_11 superfamily. The prediction of subcellular localization indicated that this protein is cytoplasmic (Reliability Index: RI = 1; Expected Accuracy = 56%). Protein signal peptide prediction revealed that the deduced signal peptide cleavage site was found between amino acids 20 and 21 (signal peptide probability: 0.781), indicating that it is a secretory protein. By transmembrane protein topological structure analysis, no transmembrane helices were detected. Motif-scan results indicated that this protein contained a casein kinase II phosphorylation site, a tyrosine kinase phosphorylation site, a protein kinase C phosphorylation site, and an N-myristoylation site, but no glycosylation sites. Amino acid composition analysis showed that this protein is rich in aspartate but poor in cysteine (Table 2), consistent with the mVg from *M*. *sexta* [7].

**Table 2:** Amino acid composition comparison among *A*. *pernyi* mVg, *M*. *sexta* mVg, and *B*. *mori* LP3.

Amino acid	*A*. *pernyi* mVg		*M*. *sexta* mVg		*B*. *mori* LP3	
	No.	%	No.	%	No.	%
**Ala (A)**	21	8.1	23	9.2	16	6.3
**Arg (R)**	16	6.2	16	6.4	10	3.9
**Asn (N) + Asp (D)**	13 + 22	13.5	14 + 20	13.6	16 + 22	14.9
**Cys (C)**	0	0	0	0	3	1.2
**Gln (Q) + Glu (E)**	9 + 19	10.8	10 + 11	8.8	10 + 15	9.8
**Gly (G)**	17	6.5	19	7.6	12	4.7
**His (H)**	6	2.3	5	2	3	1.2
**Ile (I)**	11	4.2	15	6	10	3.9
**Leu (L)**	25	9.6	21	8.4	22	8.6
**Lys (K)**	13	5	15	6	20	7.8
**Met (M)**	2	0.8	7	2.8	7	2.7
**Phe (F)**	13	5	10	4	11	4.3
**Pro (P)**	9	3.5	5	2	7	2.7
**Ser (S)**	13	5	14	5.6	16	6.3
**Thr (T)**	7	2.7	10	4	12	4.7
**Trp (W)**	6	2.3	5	2	6	2.4
**Tyr (Y)**	16	6.2	9	3.6	16	6.3
**Val (V)**	22	8.5	20	8	21	8.2

### Genomic structure

We successfully amplified a specific genomic DNA fragment using the primer pair ORF-F and ORF-R. Sequencing of the amplified genomic fragment revealed that no intron was present in the genomic DNA of this gene.

### Homologous comparison

**Fig 1:**
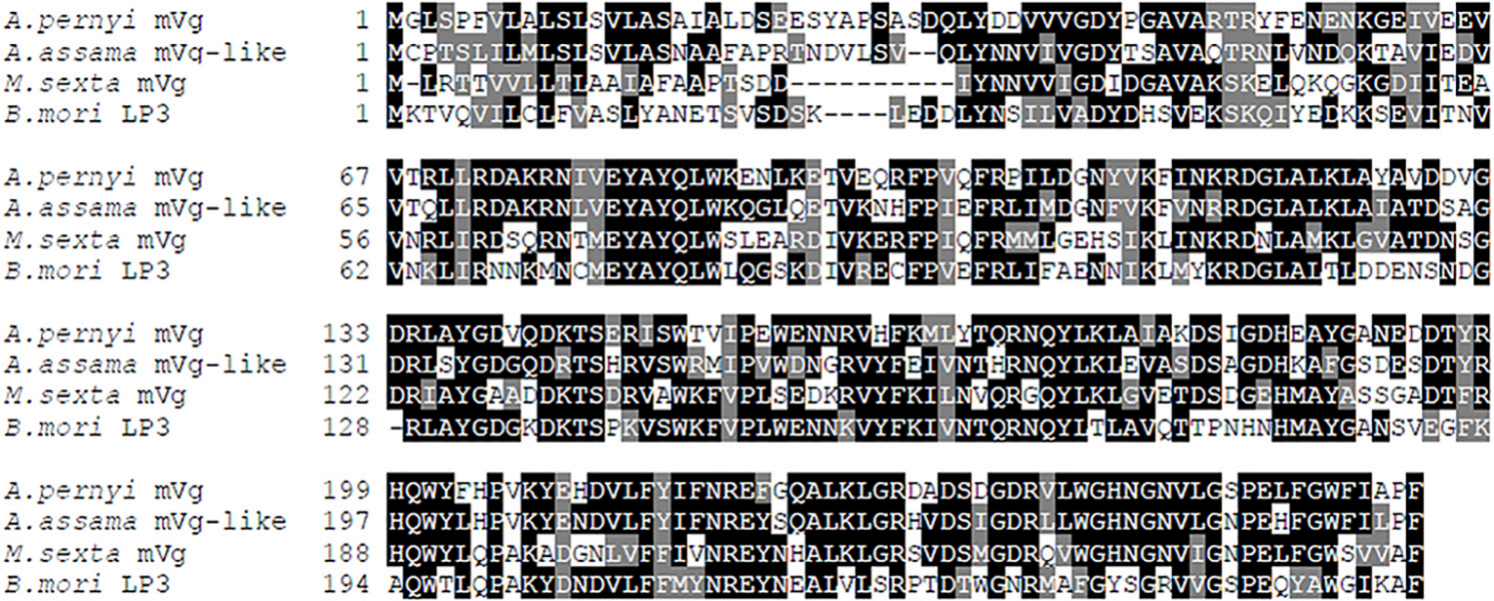
Sequence comparisons of mVgs and its homologues. These sequences include *Antheraea pernyi* mVg, *A*. *assama* mVg-like (Unigene_Aa00657 in Wildsilkbase), *Manduca sexta* mVg (AAA29342), and the *Bombyx mori* 30K protein LP3 (AFC87801). Identical amino acids are highlighted in black, and the positive amino acids are highlighted in gray.

In searching GenBank and SilkDB, a genomic database for *B*. *mori* [26], the homologues of *A*. *pernyi* mVg were only found in lepidopteran insects, including mVg from *M*. *sexta* and typical 30K proteins from *B*. *mori*. mVg in *M*. *sexta* and 30K proteins in *B*. *mori* have been studied [7,11,12]. From searches of Wildsilkbase, an EST database for wild silkworms [27], homologues were also found in *A*. *assama* (Unigene_Aa00657), *A*. *mylitta* (Unigene_Am00056), and *Samia cynthia ricini* (Sc_96Hrs2819). The related proteins from three wild silkworms have not been characterized, and only ESTs are available. Of the three proteins, only the protein from *A*. *assama* had a complete ORF available, encoding 258 amino acids with a predicted molecular weight of 29.60 kDa. Multiple sequence alignments (Fig 1) revealed that *A*. *pernyi* mVg had 64% sequence identity with the uncharacterized protein from *A*. *assama*, 48% sequence identity with mVg from *M*. *sexta*, and 40–47% sequence identity with *B*. *mori* 30K proteins.

### Phylogenetic analysis

**Fig 2:**
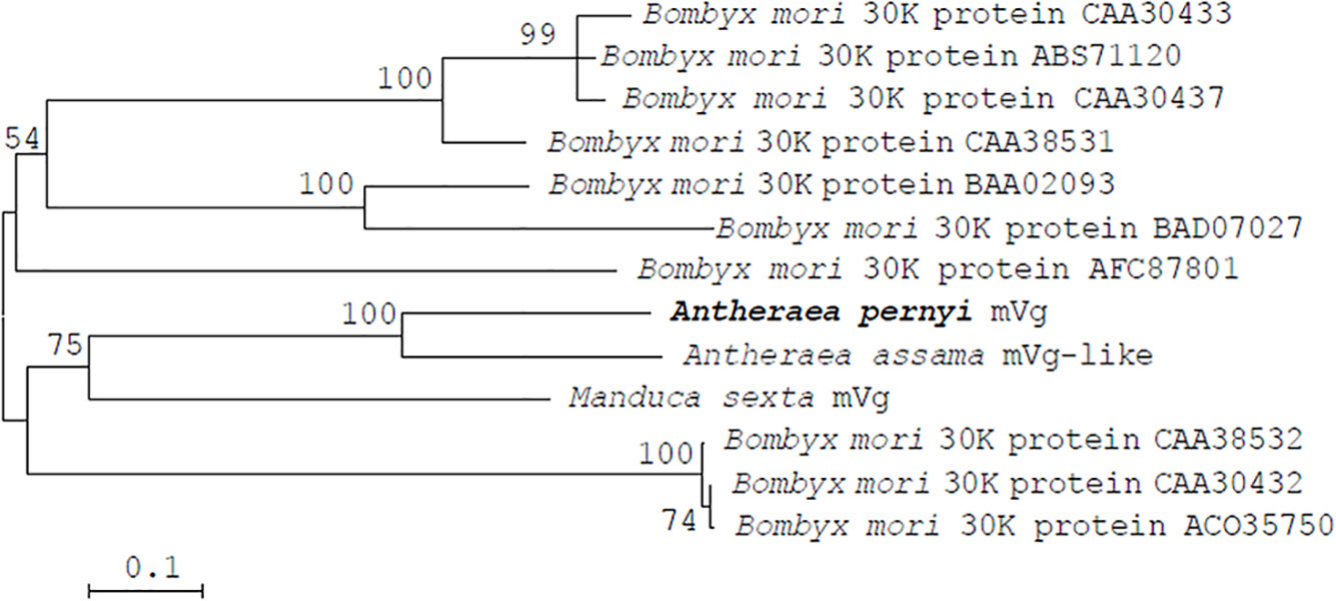
Phylogenetic tree based on amino acid sequence comparisons. The numbers near the branch represent bootstrap percentages. The topology was tested using bootstrap analyses (1,000 replicates). The GenBank accession number is shown following the organism names.

To deduce the phylogenetic relationship, a total of 13 protein sequences were considered, including *A*. *pernyi* mVg and related proteins (Fig 2). Homologous proteins from *A*. *mylitta* and *S*. *cynthia ricini* were not included, because they had incomplete protein sequences. In the ML phylogenetic tree, mVg from *A*. *pernyi* was most closely related to the mVg-like protein from *A*. *assama* with a 100% bootstrapping value; mVg from *M*. *sexta* was the next most closely related.

### mRNA expression profiles

**Fig 3:**
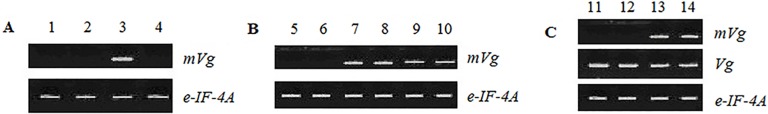
Expression of the *mVg* gene. (A) The developmental stages 1–4 represent eggs, larvae, pupae, and adults, respectively. (B) Tissue distributions in the pupal stage. The labels 5–10 indicate the brain, hemolymph (*♀*), fat body (*♀*), ovaries (*♀*), spermaries (♂), and fat body (♂), respectively. (C) The diapause stages 11–14 represent the diapausing stage, diapause-activated stage, post-diapause (1) stage, and post-diapause (2) stage, respectively. Post-diapause pupae (1) and (2) represent diapause-activated pupae without, and with, respectively, developing ovarian follicles undergoing vitellogenesis. The expression patterns were analyzed by RT-PCR using a gene-specific primer pair. The *eIF-4A* gene was used as control.

We first used RT-PCR to examine the mRNA expression profile. Sequencing the positive RT-PCR product confirmed that it was derived from the targeted gene. Investigation of the expression patterns during the four developmental stages (egg, larva, pupa, and adult) showed that the *A*. *pernyi mVg* mRNA was only expressed in the pupal stage (Fig 3A). The tissue distribution indicated that the *A*. *pernyi mVg* mRNA was specifically expressed in the fat body (female and male), spermary, or ovary (Fig 3B).

The expression changes of the *mVg* gene were investigated over four diapause stages, including diapausing, diapause-activated (5°C treatment for 50 days), post-diapause 1, and post-diapause 2 (Fig 3C). The target mRNA was not detected during the diapausing and diapause-activated stages, but it appeared in the post-diapause development stages when diapause-activated pupae without developing ovarian follicles were undergoing vitellogenesis. We also observed that the *mVg* transcript could be detected in the fat body from four days after the injection of 20-E, a stimulus for diapause termination in this species [28], and the expression level increased progressively during pupal development.

### Comparative expression patterns among three yolk protein genes

**Fig 4:**
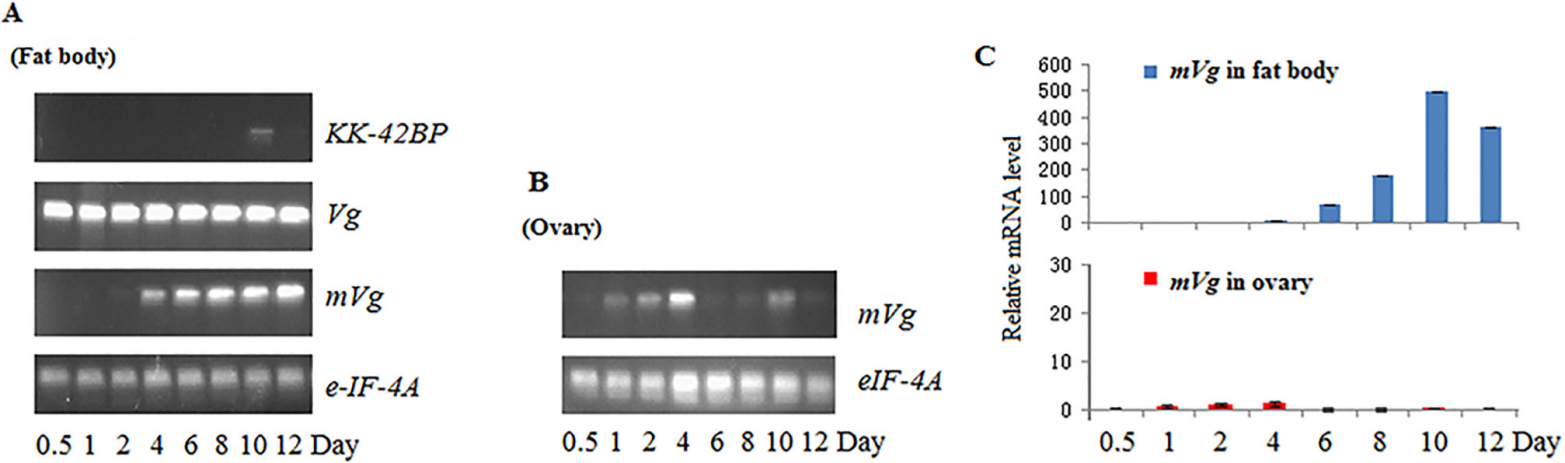
Comparative expression patterns of the *mVg* gene. (A) Expression patterns in the fat body among the *mVg*, *Vg*, and *KK-42BP* genes investigated by RT-PCR with the *eIF-4A* gene used as the control. (B) Expression of mVg in the ovary. (C) Relative mRNA expression level of the *mVg* gene in the fat body and ovary detected by qRT-PCR.

The present study demonstrated that mVg ultimately accumulates in the eggs of *A*. *pernyi* (see below). Two other yolk proteins have also been identified in the eggs of *A*. *pernyi*: Vg (the major yolk protein) and KK-42 binding protein (KK-42BP; the minor yolk protein) [20, 21]. Thus, we comparatively investigated the expression profile of the genes encoding these three yolk proteins. RT-PCR analyses revealed distinct expression patterns for these three genes (Fig 4A and 4B). *mVg* mRNA was detected on the 4^th^ day after 20-E injection, and *KK-42BP* mRNA was detected on the 10^th^ day; however *Vg* mRNA was detected in the diapausing pupae and continued to be detected during post-diapause development.

### Comparison of the transcript level between the fat body and ovary

We used qRT-PCR analysis to compare the transcript level of *mVg* between the fat body and the ovary. Our data revealed that *mVg* expression was much higher in the fat body than in the ovary (Fig 4C); approximately 600 times more *mVg* is expressed in the fat body on day 8 after 20-E treatment than in the ovaries of pupae.

### Western blot analysis

**Fig 5:**
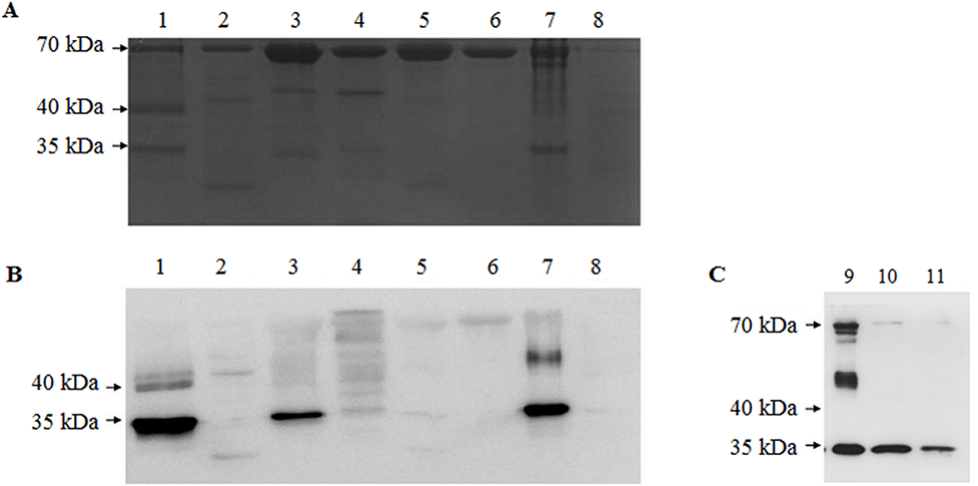
Western blot analysis of mVg in *A*. *pernyi*. (A) Coomassie blue-stained electrophoresis pattern of samples prepared from the tissues of various developmental stages and (B) Western blotting for mVg from the corresponding gel. Labels 1–8 represent eggs at day 3, larval fat body, pupal fat body (*♀*), pupal fat body (♂), pupal hemolymph (*♀*), pupal hemolymph (♂), immature eggs isolated from pupae, and moths (*♀*), respectively. (C) Western blotting for mVg from eggs during various developmental stages. Labels 9–11 represent immature eggs isolated from pupae, eggs at day 1, and eggs at day 9.

We performed Western blotting with the appropriate antibody to identify the tissues and developmental stages in which mVg is present (Fig 5A and 5B). The polyclonal antibody prepared from the synthetic 15-mer peptide reacted strongly with the samples prepared from eggs, including mature and immature eggs, and the fat bodies of female pupae, and this antibody reacted weakly with the sample from the hemolymph of female pupae or the fat bodies of male pupae. No reactions were observed with the samples prepared from the fat bodies of larvae, the hemolymph of male pupae, or female moths. We also observed that the quantity of ApmVg decreased during embryo development (Fig 5C).

## Discussion and Conclusions

To date, the molecular characteristics of mVg have been identified only in two insect species, *M*. *sexta* and *B*. *mori*. In the present study, we isolated and characterized a novel lepidopteran mVg from *A*. *pernyi*. Protein sequence comparison revealed that the characterized protein in this study shared 48% identity with the known mVg isolated from *M*. *sexta*. mRNA expression and Western blot analysis demonstrated that mVg is synthesized by the fat body, secreted into hemolymph, and ultimately accumulates in the eggs of *A*. *pernyi*, as previously described for *M*. *sexta* mVg [9]. Therefore, we identified this isolated protein as *A*. *pernyi* mVg.

Our data clearly demonstrate that *A*. *pernyi* mVg belongs to the lipoprotein_11 superfamily. Earlier studies demonstrated that the lipoprotein_11 superfamily could be divided into three subfamilies: typical 30K lipoproteins, ENF-BPs, and S/T-rich 30K lipoproteins [12]. Homology and phylogenetic analysis indicated that *A*. *pernyi* mVg is a novel member of the typical 30K lipoprotein family.

Our experiments revealed that *A*. *pernyi mVg* mRNA was expressed not only in the fat body (both female and male) but also in the ovary or spermary. Western blot analysis also demonstrated that the polyclonal antibody reacted with the samples from the fat bodies of both female and male pupae. In *A*. *assama*, a close relative of *A*. *pernyi*, *mVg*-like gene (unigene_Aa00657) ESTs were also obtained from the fat body, spermaries, and ovaries (Aafb0186, Aats1837, and Aaov2246, respectively). These results suggested that *A*. *pernyi mVg* expression is not sex specific even though its expression in males was reduced. However, by qRT-PCR analysis, we observed that *mVg* expression was 250–600 times higher in the fat body than in the ovary, indicating that the majority of the *A*. *pernyi mVg* transcripts are expressed in the fat body.

The expression patterns of genes coding for typical 30K lipoproteins are species-specific. The *M*. *sexta* mVg gene was specifically expressed in the adult stage, and the *B*. *mori* 30K protein LP3 gene was expressed in both the larval and pupal stages. The present study revealed that the *A*. *pernyi* mVg gene was specifically expressed in the pupal stage, which differs from the expression patterns of the *M*. *sexta* mVg gene and *B*. *mori* LP3 gene [7,11]. Although these genes were expressed at different stages, their products all accumulated in the eggs.

The origin of mVg is an interesting topic. The data presented here and previously reported clearly demonstrate that the mVgs from *A*. *pernyi* and *M*. *sexta* and typical 30K lipoproteins from *B*. *mori* and other Saturniidae species may be derived from a common ancestor [12]. This assumption can be explained as follows. First, they share putative conserved domains with the lepidopteran low-molecular weight lipoproteins and belong to the lipoprotein_11 superfamily. Second, they share moderate protein sequence identity (> 40%). Finally, they share similar biosynthetic processes. By RT-PCR and Western blot analysis, we conclude that *A*. *pernyi* mVg is synthesized in the fat body, secreted into the hemolymph, and transported to the developing oocytes; this journey is consistent with those of mVg from *M*. *sexta* and the 30K lipoprotein LP3 from *B*. *mori* [5,6,12].

The Western blots presented here showed that *A*. *pernyi* mVg was transported into the eggs as yolk protein, and the amount of mVg in eggs decreased during the embryo development. These results suggested that *A*. *pernyi* mVg could play a role in silkworm hatching by acting as a storage protein, as has been observed for the 30K proteins LP1-4 in *B*. *mori* [29,30] and mVg in *M*. *sexta* [9]. Our motif-scan analyses revealed that *A*. *pernyi* mVg contains three protein kinase phosphorylation sites, including casein kinase II, tyrosine kinase, and protein kinase C. These three protein kinase phosphorylation sites are also observed in *B*. *mori* LP3 [11]. A recent study has shown that the *B*. *mori* LP3 is degraded in the embryonic development by a specific proteolytic pathway [12]. Therefore, we envision that *A*. *pernyi* mVg is also degraded by the specific proteolytic pathway previously predicted for *B*. *mori* LP3 [11,12].

Previous studies based on immunization have demonstrated that *A*. *pernyi* Vg synthesis by the fat body starts in female larvae at the time of the spinning stage [31,32] and continues during the whole diapause stage and through the pupal-adult developmental stage [31]. The present study provides evidence at the transcriptional level that the *Vg* gene is expressed throughout four diapause developmental stages, indicating an expression pattern distinct from that of the *mVg* gene, which is specifically expressed in the post-diapause pupal stage. Our data further confirmed that mVg and Vg can be internalized via separate receptor systems [6]. Our results also revealed distinct expression patterns between the *mVg* and *KK-42BP* genes, although both were expressed in the post-diapause pupal stage. Moreover, we did not observe any protein sequence homology between KK-42BP and mVg, indicating that they are not derived from a common ancestor. The distinct expression patterns among the three yolk protein genes suggest that they play different roles in egg development.

In conclusion, the present study reports a novel mVg from *A*. *pernyi*. ApmVg belongs to typical 30K proteins within the lipoprotein_11 superfamily. The *ApmVg* mRNA is only detected in the pupal stage but not in a sex-specific manner. ApmVg is synthesized by the fat body, secreted into hemolymph, and ultimately accumulates in the eggs. ApmVg decreases dramatically during embryonic development. We also observed distinct expression patterns between the *mVg* and *Vg* genes and between the *mVg* and *KK-42BP* genes. The data presented here contribute to the understanding of the physiological role and origin of mVg in insects.

## Supporting Information

S1 Fig The nucleotide and deduced amino acid sequences of the *A*. *pernyi mVg* gene. The cDNA (928 bp) contains a complete ORF encoding a protein of 260 amino acid residues. This cDNA sequence has been deposited in GenBank under accession no. KM926620. The initiation codon ATG is in bold, and the termination codon TAA is in bold and marked with an asterisk. The polyadenylation signal AATAA is double-underlined. **@**, Signal peptide site; **#**, casein kinase II phosphorylation site; **$**, tyrosine kinase phosphorylation site; **%**, protein kinase C phosphorylation site; **&**, N-myristoylation site.
(PDF)
