# Supplementary material for: Identification and Characterization of a Novel Microvitellogenin from the Chinese Oak Silkworm Antheraea pernyi
Source: PLoS One. 2015 Jun 30;10(6):e0131751. doi: 10.1371/journal.pone.0131751 (PMC4488348; doi:10.1371/journal.pone.0131751)
Supplement: S1 Fig — The cDNA (928 bp) contains a complete ORF encoding a protein of 260 amino acid residues. This cDNA sequence has been deposited in GenBank under accession no. KM926620. The initiation codon ATG is in bold, and the termination codon TAA is in bold and marked with an asterisk. The polyadenylation signal AATAA is double-underlined. @, Signal peptide site; #, casein kinase II phosphorylation site; $, tyrosine kinase phosphorylation site; %, protein kinase C phosphorylation site; &, N-myristoylation site. (PDF) [file pone.0131751.s001.pdf]

```
1                                               AACTACTACTCGTGGTTTATTCAGGTAGAAGTGTCTCAAA
41     ATGGGCCTGTCGCCTTTCGTCCTAGCGCTATCTCTGAGCGTGCTAGCGTCCGCCATCGCACTGGATTCAGAAGAA
        M  G  L  S  P  F  V  L  A  L  S  L  S  V  L  A  S  A  I  A  L  D  S  E  E
                                    @
116    AGCTACGCACCCTCGGCTTCAGACCAACTATACGACGATGTGGTCGTTGGGGACTACCCTGGCGCTGTGGCTCGG
        S  Y  A  P  S  A  S  D  Q  L  Y  D  D  V  V  V  G  D  Y  P  G  A  V  A  R
                       #
191    ACACGCTATTTCGAAAACGAAAACAAAGGCGAAATAGTCGAAGAAGTTGTAACCAGACTACTGAGAGACGCGAAA
        T  R  Y  F  E  N  E  N  K  G  E  I  V  E  E  V  V  T  R  L  L  R  D  A  K
266    CGGAACATAGTCGAGTACGCTTACCAATTGTGGAAAGAAAACCTAAAGGAAACAGTCGAACAGCGTTTTCCAGTA
        R  N  I  V  E  Y  A  Y  Q  L  W  K  E  N  L  K  E  T  V  E  Q  R  F  P  V
              $
341    CAATTCCGGCCTATTTTGGACGGAAACTATGTCAAGTTTATCAACAAGAGAGACGGTTTAGCACTAAAACTCGCG
        Q  F  R  P  I  L  D  G  N  Y  V  K  F  I  N  K  R  D  G  L  A  L  K  L  A
                     $
416    TACGCAGTTGACGATGTGGGAGATAGGTTGGCGTACGGTGACGTACAGGATAAAACTAGCGAACGAATCAGCTGG
        Y  A  V  D  D  V  G  D  R  L  A  Y  G  D  V  Q  D  K  T  S  E  R  I  S  W
                                                                    %
491    ACCGTGATCCCGGAGTGGGAGAACAACAGGGTGCATTTTAAAATGCTGTACACCCAGCGGAATCAATACTTGAAG
        T  V  I  P  E  W  E  N  N  R  V  H  F  K  M  L  Y  T  Q  R  N  Q  Y  L  K
                                                                %
566    TTGGCCATAGCTAAGGACAGCATCGGAGATCACGAAGCGTATGGTGCCAATGAAGATGATACTTACAGACATCAG
        L  A  I  A  K  D  S  I  G  D  H  E  A  Y  G  A  N  E  D  D  T  Y  R  H  Q
                             #                                         %
641    TGGTACTTCCACCCGGTGAAGTACGAGCATGACGTGTTGTTCTATATCTTCAACCGTGAGTTCGGTCAGGCTCTG
        W  Y  F  H  P  V  K  Y  E  H  D  V  L  F  Y  I  F  N  R  E  F  G  Q  A  L
                                      $
716    AAGCTAGGCAGAGACGCTGACAGTGATGGGGACCGCGTTCTGTGGGGGCATAACGGCAATGTTCTTGGTAGCCCC
        K  L  G  R  D  A  D  S  D  G  D  R  V  L  W  G  H  N  G  N  V  L  G  S  P
                                #                                   &
791    GAGTTGTTTGGATGGTTTATCGCACCTTTCTAAAATAAATAACTCGGATATTTCGAACCATGACAAATGAATGAA
        E  L  F  G  W  F  I  A  P  F  *
866    TATTTTGATAATACATGTTTTAAAAATCAAAAAAAAAAAAAAAAAAAAAAAAAAAAAAAAAAA
```
